# Prognostic and Therapeutic Roles of the Insulin Growth Factor System in Glioblastoma

**DOI:** 10.3389/fonc.2020.612385

**Published:** 2021-02-02

**Authors:** Elena Tirrò, Michele Massimino, Chiara Romano, Federica Martorana, Maria Stella Pennisi, Stefania Stella, Giuliana Pavone, Sandra Di Gregorio, Adriana Puma, Cristina Tomarchio, Silvia Rita Vitale, Livia Manzella, Paolo Vigneri

**Affiliations:** ^1^ Department of Clinical and Experimental Medicine, University of Catania, Catania, Italy; ^2^ Center of Experimental Oncology and Hematology, A.O.U. Policlinico “G. Rodolico—San Marco”, Catania, Italy; ^3^ Medical Oncology, A.O.U. Policlinico “G. Rodolico—San Marco”, Catania, Italy

**Keywords:** insulin/insulin-like growth factor system, insulin-like growth factor signaling pathway, IGF-binding protein, glioblastoma, drug resistance

## Abstract

Glioblastoma multiforme (GBM) is the most common primary brain malignancy and is often resistant to conventional treatments due to its extensive cellular heterogeneity. Thus, the overall survival of GBM patients remains extremely poor. Insulin-like growth factor (IGF) signaling entails a complex system that is a key regulator of cell transformation, growth and cell-cycle progression. Hence, its deregulation is frequently involved in the development of several cancers, including brain malignancies. In GBM, differential expression of several IGF system components and alterations of this signaling axis are linked to significantly worse prognosis and reduced responsiveness to temozolomide, the most commonly used pharmacological agent for the treatment of the disease. In the present review we summarize the biological role of the IGF system in the pathogenesis of GBM and comprehensively discuss its clinical significance and contribution to the development of resistance to standard chemotherapy and experimental treatments.

## Introduction

Malignant gliomas represent 30% of all intracranial tumors and include a heterogeneous group of neoplasms that arise from multiple cell types with neural stem cell-like properties (1). In the US, the annual incidence of gliomas is 3.2 cases for every 100,000 individuals and about half of them are classified as glioblastoma or glioblastoma multiforme (GBM) (2). Incidence increases with age and is more common in men than in women and in individuals of caucasian ethnicity (3). Disease prognosis is poor, with a 35% survival rate at 1 year and <5% at 5 years (4). GBM can be classified as primary when it develops within a few months without known precursor states, or secondary, when a low-grade tumor evolves in a GBM. Surgery, radiation therapy and chemotherapy with alkylating agents such as temozolomide (TMZ) represent the mainstay of GBM treatment (5). Additionally, several small molecules have been tested in the last few years (6, 7). However, none of these strategies represent an effective cure for GBM, as this malignancy displays extensive intra-tumoral heterogeneity that favors disease recurrence (8, 9). Genomic profiling carried out by the The Cancer Genome Atlas (TCGA) consortium on 200 GBM samples as well as a complementary study by Pearson and colleagues revealed recurrent genetic alterations involving *TP53*, retinoblastoma (*Rb*) and different receptor tyrosine kinases (RTK) pathways (10, 11).

RTKs are a family of cell surface receptors, comprising the epidermal growth factor receptor (EGFR), the fibroblast growth factor receptor (FGFR), the hepatocyte growth factor receptor (HGFR/c-MET), the platelet-derived growth factor receptor (PDGFR), the vascular endothelial growth factor receptor (VEGFR) and the insulin-like growth factor 1 receptor (IGF-1R). The latter is part of the insulin and insulin-like growth factor (IGF) family that includes three ligands (insulin, IGF-I and IGF-II), different cell surface receptors (the insulin receptor, the IGF-IR, IGF-IIR and hybrid heterodimer receptors between the insulin receptor and IGF-IR) and six IGF-binding proteins (IGFBP 1 to 6). This complex system promotes the release of IGFs modulating their interaction with receptors and different IGFBP proteases (12–15).

In the extracellular compartment, insulin and IGFs activate intracellular signaling pathways by binding—with different affinity—their cognate and/or non-cognate receptors. The interaction between ligands and receptors results in the recruitment of downstream insulin receptor substrates (IRSs) and SRC homologous and collagen-like (SHC) proteins. These cytoplasmatic proteins modulate the activation of the PI3K/AKT/mTOR axis and the RAS/RAF/MEK/ERK pathway involved in the transcription of genes regulating cell proliferation, cell-cycle progression, cell motility and apoptosis (16).

Aberrant activation of the Insulin/IGF signaling plays a crucial role in dysregulation of multiple cellular pathways in different tumors and its atypical activation is usually associated with a poor prognosis (17–21).

In the present review we summarize current research on the role of the IGF system in the pathogenesis of GBM and discuss the clinical significance and therapeutic implications of this pathway in the development of resistance to both currently approved and experimental treatments.

## Expression of IGF System Components in GBM

Studies on the expression of Insulin/IGF system components in human GMB samples are often conflicting and depend on the detection technique employed.

Use of *in situ* hybridization (ISH) or immunohistochemistry (IHC) techniques for the detection of IGF-I and II revealed high levels of these two ligands in GBM samples compared to normal glial tissue (22–25). On the contrary, analyses conducted by real-time PCR on tissue microarrays showed no change in IGF-I or IGF-II transcripts between normal glial samples and high-grade gliomas, including GBMs (23, 25).

Both IGF-IR and IGF-IIR are overexpressed in GBMs compared with normal brain and this overexpression is often associated with inferior survival and a less favorable response to therapy (23, 26).

IGFBPs overexpression is also well documented in glioblastoma. Different studies demonstrate that IGFBP-2 is overexpressed in glioma cells showing a distinct progression-related expression change from low- to high-grade gliomas (27–29). Indeed, high IGFBP-2 levels promote both proliferation and invasion of glioma cells and have been linked to an adverse prognosis in high-grade gliomas (30–32).

IGFBP-3, IGFBP-4 and IGFBP-5 transcripts are significantly higher in GBM compared to low-grade gliomas or normal samples, supporting their role in the pathogenesis of gliomas. Furthermore, IGFBP-4 up-regulation favors epithelial-mesenchymal transition (EMT), proliferation, migration and invasion (29, 33, 34). Unlike other IGFBPs, the expression of IGFBP-6 inversely correlates with glioma grade and higher plasma IGFBP-6 levels have been associated with a better prognosis (15, 35).

Therefore, dysregulation of several IGF system components is usually associated with a more aggressive disease displaying poor response to therapy and a shorter survival (15).

## The IGF Signaling Pathway in GBM

**Figure 1:**
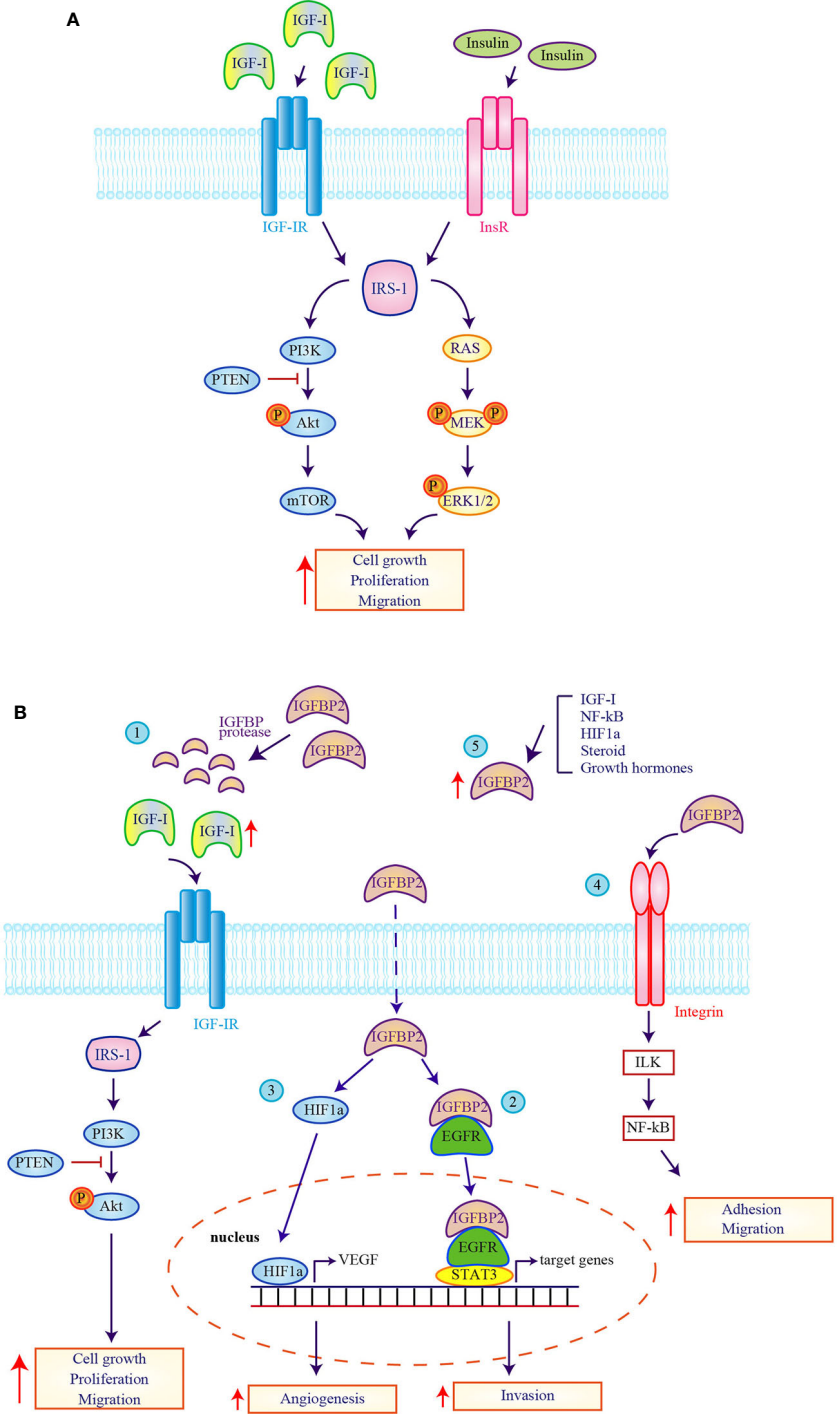
Expression and function of the insulin/insulin-like growth factor system in glioblastoma cells. **(A)** The pathogenesis and progression of glioblastoma multiforme (GBM) is mainly linked to the activation of Insulin-like growth factor 1 receptor (IGF-IR) and insulin receptor (InsR). The stimulation of IGF-IR and the activation of InsR promote the recruitment of IRS-1 and the activation of the mitogenic and pro-survival mediators Akt and ERK1/2 that contribute to increase (upward red arrow) GBM cell growth, cell proliferation and cell migration. **(B)** A pivotal role in regulating IGF-system action in GBM pathogenesis is provided by IGFBP-2. (1) The improper activity of protease that cleaves IGFBP-2 causes an increase of circulating-free IGFs (upward red arrow) and the subsequent activation of downstream pathway causing an increase on cell growth, cell proliferation and cell migration. The intracytoplasmatic and intranuclear activity of IGFBP-2 determines the activation of EGFR-STAT-3 (2) or HIF1a (3) signaling and the improvement of the transcription of their target genes. IGFBP-2 activates also the integrin signaling (4) which lead to the activation of the IGFBP-2/Integrin/ILK/NF-kB pathway that is responsible of the transcription of genes linked to adhesion and migration. Finally, stimuli induced by IGF-I, NF-kB, HIF1a, steroid and growth hormones (5) increase IGFBP-2 expression (upward red arrow) causing the activation of pathway linked to GBM progression.

IGF-I interaction with IGF-IR appears to trigger low-grade glioma progression to GBM (**Figure 1A**). Specifically, IGF-IR stimulation by IGF-I promotes glioma cell proliferation and migration by negatively or positively modulating PI3K/AKT signaling through a mechanism conditioned by a specific cellular context (36, 37). In a subpopulation of glioma stem cell-like cells identified as recurrence-initiating stem-like cancer (RISC) cells, IGF-IR maintains cell survival through an autocrine activation that downregulates both AKT and ERK signaling leading to slow-growth but high-self-renewal (38, 39). On the contrary, this mechanism is not observed in non-glioma stem cells. Furthermore, Hagerstrand and colleagues demonstrated that glioma cells showed ligand-independent AKT phosphorylation and that combined inhibition of IGF-IR and PI3K or mTOR reduces cell viability (37). These data are also supported by recent evidence suggesting that, IGF-I or IGF-IR overexpression causes AKT phosphorylation while its inhibition reduces IGF-mediated anti-apoptotic effects (40).

Involvement of the AKT pathway in GBM pathogenesis is also connected to activation of the insulin receptor (InsR) that promotes proliferation and survival of glioma cells both in an insulin-dependent and -independent manner (**Figure 1A**). Insulin signaling is robustly activated in human glioma cells independently of insulin stimulation, since the catalytic subunit of the InsR strongly phosphorylates and recruits IRS-1 leading to the activation of AKT and ERK2 (41) (**Figure 1A**). Furthermore, experiments conducted in insulin-stimulated IRS-1-transfected glioma cell showed high phosphorylation levels of AKT, ERK1/2 and overexpression of Grb2 resulting in increased cell viability (42).

The compensatory mechanism within the InsR/IGF signaling network was widely described in different tumor types (43, 44). Gong and colleagues found predominant InsR mitogenic isoform-A in GBM surgical specimens and xenograft tumor lines. Interestingly, they observed that InsR-A depletion compromised Akt activation repressing orthotopic tumor growth, but this event has been restored by stimulated IGF-IR expression. These data confirm the cooperation between InsR and IGF-IR also in glioma cells, suggesting a compensatory crosstalk addressed to balance potential defective activity in one of two signaling (45).

Crosstalk between InsR/IGF systems and other RTK pathways has been demonstrated in many human cancers, including GBM (46–50). It was reported that Hedgehog-IGF-I crosstalk preserves the self-renewal properties in GBM. This interaction causes VEGF expression and increases survival and proliferation in Glioma Stem Cell (GSC) involving Gli1 but not Gli2 proteins (51, 52). A crosstalk between InsR/IGF-IR and EGFR linked to an Akt-mediated compensatory intracellular mechanism was also described (53).

IGFBPs have multiple and complex functions playing a pivotal and significant role in regulating IGF-system action in GBM. Different authors reported IGFBP-2 as central mediators in GBM pathogenesis and two controversial mechanisms have been hypothesized (**Figure 1B**). The first one includes an improper protease activity of IGFBP-2 that determines an increase of circulating-free IGFs and the activation of EGFR-STAT3 signaling in an IGF-independent manner, involving the intracytoplasmic and intranuclear IGFBP2 functions (54). The second mechanism is more complex. In GBM cells IGFBP-2 expression is inversely correlated with PTEN levels, thus promoting Akt-mediated signaling. In addition, stimuli induced by IGF-I, NF-kB, hypoxia-inducible factor 1a (HIF1a), steroid and growth hormones increase IGFBP-2 expression eventually causing GBM progression. Furthermore, increased IGFBP-2 levels have been directly associated to enhanced cell proliferation and correlates with HIF1a-mediated stimulation of VEGF pathway (55). Moreover, IGFBP-2 and integrin alpha5 interaction seem necessary to promote glioma cell migration in a JNK-dependent manner (56). Finally, IGFBP-2 was also identified as critical component of a complex network (IGFBP-2/Integrin/Integrin-Linked Kinase (ILK)/NF-kB) able to drive glioma progression. Kristen and colleagues proposed that IGFBP-2 is request for the formation of integrin complex which lead to ILK and NF-kB activation responsible of diffuse glioma progression (27).

As well as IGFBP-2, also IGFBP-3 improves glioma cell migration, invasion and proliferation in an IGF-independent manner. The tumor promoting properties of IGFBP-3 are linked to its ability to increase STAT-1 expression that, in turn, result associated with reduced patients overall survival (OS) (57). Furthermore, Chia-Hua Chen demonstrated that IGFBP-3 silencing suppresses glioma cell proliferation by G2/M cell cycle arrest (58). On the contrary, the anti-tumorigenic and anti-angiogenetic functions of IGFBP-4 in glioma cells depend to its ability to induce dibutyryl cyclic AMP (dB-cAMP) that antagonize the VEGFs action in an IGF-independent manner (59).

IGFBP-5 is highly expressed in GBM, indeed its depletion results in an inhibition of cell invasion and concomitant increase in cell proliferation. The double role of IGFBP-5 is mechanistically associated with Akt and EMT signaling (60).

Lastly, IGFBP-6 is a member of a paracrine signaling circuit involving the IGF-II-IGF-IR axis present in TMZ-resistant glioma cells. IGFBP-6, released by TMZ-sensitive cells, reduces the expansion of the counterpart resistant cell population by sequestering IGF-II that results in IGF-IR-Akt signaling inactivation (15).

## MicroRNAs as Regulators of the IGF System in GBM

MicroRNAs (miRNAs) are a class of small non-coding RNAs that are usually classified into tumor-suppressive miRNAs (TS-miRNAs) and onco-miRNAs depending on their ability to suppress or favor tumorigenesis. MiRNA dysregulation is associated with initiation and progression of several forms of cancer, including GBM (61, 62).

It has been reported that miRNA miR-128, miR-422a and miR-603 inhibit IGF-I expression in glioma cells as overexpression of these miRNAs suppresses cell proliferation, migration and invasion (63) and enhances apoptotic death through the inhibition of the mTOR signaling pathway (64). Furthermore, a plethora of TS-miRNAs (including miR-7, miR-15b, miR-181b, miR-186, miR-320a, miRNA-323-5p, miR-383, miR-422a, miR-503, miR-505, and miR603) target IGF-IR messenger RNA in the non-neoplastic brain, thereby acting as tumor-suppressors. In GBM the expression of these miRNAs is reduced resulting in activation of the IGF-IR/AKT axis. *In vitro* studies demonstrated that TS-miRNAs overexpression also induces cell-cycle arrest and apoptosis (63, 65–74). Several miRNAs modulate IGF/IGF-IR signaling by targeting its downstream effectors. Indeed, miR-204-3p and miR-491-3p inhibit IGFBP-2 expression and are downregulated in glioma cells (75, 76). MiR-302b indirectly inhibits IGFBP-2 activity by downregulating the expression of its direct target Nuclear factor IA (NFIA), which transcriptionally regulates IGFBP-2 expression (77). MiR‐7 potently inhibits EGFR, IRS‐1 and IRS‐2 expression (78), while the brain-specific miR-153 downregulates IRS-2 (79). Moreover, the tumor suppressor miR-128-3p indirectly regulates IRS−1 expression by modulating the expression of Neuronal Pentraxin 1 situated upstream of the IRS−1/PI3K/AKT pathway (80). The reported downregulation of these TS-miRNAs in malignant glioma results in IRS overexpression and activation of the PI3K/AKT pathway (78, 79).

Finally, among the onco-miRNAs, only miR-21 and miR-513a-5p are overexpressed in human GBM cell lines and tumor tissue (81, 82). Yang and colleagues reported that high levels of miR-21 lead to downregulation of IGFBP-3, restraining its antiproliferative activity. Interestingly, miR-21 expression levels are inversely correlated with GBM survival (82).

## The IGF System and Treatment Resistance in GBM

Several pre-clinical evidences suggest that the IGF system plays a pivotal role in the development of resistance to chemotherapy, radiation and targeted therapies, eventually resulting in GBM recurrence and/or progression (83).

Differences in both protein expression and miRNA production related to the IGF pathway have been found in TMZ-sensitive and -resistant GBM cells. These differences could be exploited to restore chemo-sensitivity in resistant cells. Indeed, compared with TMZ-sensitive malignant glioma cells, the resistant ones express higher miR-497 levels. This difference may play a role in the induction of TMZ resistance through the activation of the IGF-IR/IRS-1 pathway-related proteins, that are IGF-IR, IRS1, mTOR and Bcl-2 (84). Furthermore, TMZ-resistant cells display lower IGFBP-6 expression than TMZ-sensitive cells, with the latter down-regulating both IGF-II and IGF-IR as IGFBP-6 acts as a suicide substrate binding IGF-II with high affinity and preventing its interaction with IGF-IR. Although the site of IGFBP-6 endogenous production remains unclear, this model suggests a paracrine secretion from TMZ-sensitive cells. Hence, by depleting the sensitive population, TMZ selects the resistant one, ultimately promoting growth of the tumor mass (15, 84). The IGF-system also seems to determine resistance to alkylating agents through a miRNA-mediated activation of WNT/β-catenin signaling. In detail, IGF-I up-regulates miR-513a-5p, which in turn represses NEural precursor cell-expressed Developmentally Downregulated 4-Like (NEDD4L), ultimately leading to WNT/β-catenin activation (81). *NEDD4L* is a tumor suppressor gene encoding for a ubiquitine ligase, which regulates ion channel expression and intracellular signaling (85). Indeed, low NEDD4L levels correlate with TMZ resistance and poor prognosis in gliomas (86).

The induction of a stem-cell phenotype is one of the main causes of resistance to radiation therapy in GBM. IGF signaling is involved in this process trough mechanisms only partially understood (39, 87). MiR-603 targets IGF-I and IGF-IR, facilitating the exit from the stem-cell condition and conferring sensitivity toward radiation. However, ionizing radiation reduces miR-603 expression by enhancing its extracellular vesicle-mediated export. Hence, lower levels of MiR-603 induced by radiotherapy may promote a stem-cell state. miR-603 also suppresses O6-MethylGuanine-DNA-MethylTransferase (MGMT). Consequently, decreased miR-603 activity determines MGMT up-regulation, which ultimately leads to cross-resistance toward alkylating agents in glioma cells (74). Recently, Simpson et al. further supported the involvement of the IGF-IR in the radiation resistance of high grade gliomas describing radio-sensitization after IGF-IR inhibition in pediatric patients (88).

Even though the EGFR represents a potential target for GBM treatment, its inhibition led to dismal results due to intrinsic tumor resistance to this approach (89, 90). Indeed, activation of a compensatory IGF pathway exerts a pivotal role in the lack of sensitivity toward EGFR inhibition. Simultaneous targeting of the EGFR and IGF-IR was effective in GBM cell lines and patient derived xenografts (PDX) (53, 91). On a different note, miR-7 overexpression sensitizes GBM cells to the antitumor effect of erlotinib *via* blockage of the IRS/AKT pathway (92). Thus, quantification of IGF-IR tissue levels may be employed as a predictive biomarker to improve the selection of GBM patients likely to benefit from EGFR inhibitors as reported in the NCT00897663 trial.

The PDGFR is another RTK involved in gliomas development. As for EGFR, PDGFR inhibition has marginal activity in GBM (89, 93, 94). Again, the InsR and IGF-IR seem key effectors of resistance to PDGFR inhibitors and combining an anti-PDGFR and an anti IGF-IR decrease the viability of primary mouse tumor sphere PDGFR-resistant clones obtained from glioma samples *in vitro* (95).

Inhibition of key components within the GBM tumor micro-environment may also represent an attractive strategy. To this end, colony-stimulating factor-1 receptor (CSF-1R) inhibitors, which block the tumor-associated macrophages and microglia, are currently under development (96, 97). Quail and colleagues investigated the mechanism of resistance to CSF-1R inhibition in murine models of GBM, discovering that it is mediated by activation of the PI3K pathway *via* the IGF-I/IGF-IR system. Hence, coupling CSF-1R and PI3K or IGF-IR inhibition elicits a sustained response in mice (98).

Recently, a molecule targeting the sarcoendoplasmic reticulum Ca2+ ATPase (SERCA) has been tested on patient-derived GBM neurospheres, identifying responder and non-responder cell lines. The latter seem to bypass SERCA activity *via* a mechanism mediated by IRE1 (derived from *ERN1* gene), IGFBP-3 and IGFBP-5. Indeed, CRISPR-mediated deletion of the *ERN1, IGFBP3, IGFBP5* genes in a human GBM cell line increased responsiveness to drugs (99).

## Anti-IGF Therapeutic Strategies in GBM

Despite the many advances in the field of molecular targeted therapy, the use of small molecules agents or monoclonal antibodies for the treatment of GBM is extremely limited and some promising pre-clinical results have yet to translate into meaningful therapeutic benefits (100–103).

**Figure 2:**
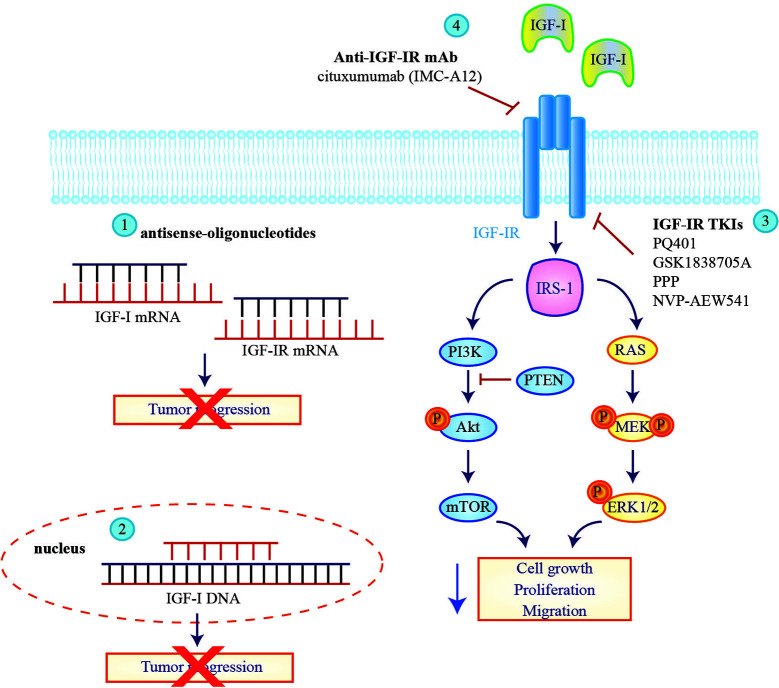
Therapeutic strategies to block insulin-like growth factor signaling in glioblastoma multiforme cells. Approaches tested to inhibit insulin-like growth factor (IGF) signaling in glioblastoma (GBM) patients include antisense oligonucleotides against IGF-I and IGF-IR mRNA (1) or the employ of IGF-I triple helix gene therapy (2) to induce a block of tumor progression. IGF-IR direct inhibitors, IGF-IR TKIs (3) and IGF-IR mAbs (4) are also tested on GBM cell lines to reduce IGF downstream signaling.

Many approaches have been tested to inhibit IGF signaling in GBM including anti-sense oligonucleotides, tyrosine kinase inhibitors (TKIs) or monoclonal antibodies targeting the IGF-IR (**Figure 2**). In a pilot study, inhibition of IGF-IR expression by antisense strategy failed to produce any clinical benefit for patients with recurrent GBM (104). An improvement was obtained by employing the combination of IGF-I antisense/triple helix vector strategy to create immunogenic cells that were injected in GBM patients where they induced an antitumor immune response and stopped tumor progression. The survival of two treated GBM patients reached 2 years (105, 106).

Promising results were also obtained with a treatment based on autologous glioma cells treated *ex vivo* with an antisense oligodeoxynucleotide targeting the IGF-IR (IGF-1R/AS ODN) and re-implanted in patients. Seventeen out of 33 individuals enrolled in a phase Ib trial (NCT02507583) remained progression-free and 12 of them are still alive, demonstrating that an autologous cell vaccine may significantly prolong progression free survival (PFS) and OS compared to standard of care (107).

Several IGF-IR-directed TKIs have been tested in GBM patients with promising pre-clinical results. PQ401, GSK1838705A and PPP (picropodophyllin/AXL1717) all reduced cell viability and proliferation *in vitro*, while the administration in mice led to inhibition of glioma tumor growth *in vivo* (26, 108, 109). Notably PPP causes dramatic tumor regression not only in subcutaneous but also in intracerebral xenograft models, indicating that it crosses the blood-brain barrier. Hence a phase I/II clinical trial was initiated (NCT101721577) but results have not yet been posted. NVP-AEW541 is another IGF-IR-specific TKI that in combination with dasatinib significantly increases BAX activation inducing more apoptosis than either agent alone in glioma cells (110).

Multiple IGF-IR blocking antibodies are currently in clinical development (111) but - to date - only cixutumumab (IMC-A12) has been clinically tested on patients diagnosed with GBM. Cixutumumab binds IGF-IR or IGF-IR/IR hybrid receptors blocking the interaction with their ligands and inducing receptor internalization and degradation. Zamykal and colleagues demonstrated that cixutumumab inhibits GBM progression in *in vivo* xenograft models (112). A phase I study of IMC-A12 in combination with temsirolimus was also conducted in a group of pediatric patients including four with refractory GBM demonstrating that this combination was well tolerated (113). However these results were deemed insufficient to pursue further development of this compound.

## Conclusions

GBM is an aggressive disease, with a dismal prognosis and scarce therapeutic resources. Hence, understanding the biological features underlying disease pathogenesis, maintenance and progression represents urgent and unmet medical need. To date, a plethora of pre-clinical evidence demonstrates a crucial role for the IGF system in GBM. Altered IGF signaling as well as cross-talk with multiple pathways are complex and far from being fully understood. However, the IGF system could potentially be exploited for both diagnostic and therapeutic purposes. For example, the expression level of many components of the IGF signaling axis or their serum concentration in GBM patients may become prognostic biomarkers, eventually guiding therapeutic choices. Unfortunately, thus far the clinical efficacy of treatment strategies directed against the IGF system remains limited and inconsistent. Inter- and intra-tumor heterogeneity, cross talk between IGF and RTK signaling and limited drug distribution to the central nervous system, are some of the potential reasons for the lack of substantial anti-cancer activity. Hence, further development of innovative therapeutic approaches is warranted in order to expand the therapeutic armamentarium for GBM patients.

## Author Contributions

Conceptualization, ET and MM. Formal analysis, ET and SG. Data curation, ET and CT. Writing—original draft preparation, ET, MM, MP, SS, FM, GP, CR, AP, and LM. Writing—review and editing, ET, MM, MP, SS, FM, AP, GP, CR, SV, and LM. Supervision, PV. All authors contributed to the article and approved the submitted version.

## Conflict of Interest

The authors declare that the research was conducted in the absence of any commercial or financial relationships that could be construed as a potential conflict of interest.

## References

[1] Weller M Wick W Aldape K Brada M Berger M Pfister SM Glioma. Nat Rev Dis Primers (2015) 1:15017. 10.1038/nrdp.2015.17 27188790

[2] Ostrom QT Cote DJ Ascha M Kruchko C Barnholtz-Sloan JS Adult Glioma Incidence and Survival by Race or Ethnicity in the United States From 2000 to 2014. JAMA Oncol (2018) 4(9):1254–62. 10.1001/jamaoncol.2018.1789 PMC6143018 29931168

[3] Davis ME Stoiber AM Glioblastoma multiforme: enhancing survival and quality of life. Clin J Oncol Nurs (2011) 15(3):291–7. 10.1188/11.CJON.291-297 21624864

[4] Ostrom QT Gittleman H Farah P Ondracek A Chen Y Wolinsky Y CBTRUS statistical report: Primary brain and central nervous system tumors diagnosed in the United States in 2006-2010. Neuro Oncol (2013) 15 Suppl 2:ii1–56. 10.1093/neuonc/not151 24137015 PMC3798196

[5] Tan AC Ashley DM Lopez GY Malinzak M Friedman HS Khasraw M Management of glioblastoma: State of the art and future directions. CA Cancer J Clin (2020) 70(4):299–312. 10.3322/caac.21613 32478924

[6] Kim G Ko YT Small molecule tyrosine kinase inhibitors in glioblastoma. Arch Pharm Res (2020) 43(4):385–94. 10.1007/s12272-020-01232-3 32239429

[7] Lee A Arasaratnam M Chan DLH Khasraw M Howell VM Wheeler H Anti-epidermal growth factor receptor therapy for glioblastoma in adults. Cochrane Database Syst Rev (2020) 5:CD013238. 10.1002/14651858.CD013238.pub2 32395825 PMC7389448

[8] Phillips HS Kharbanda S Chen R Forrest WF Soriano RH Wu TD Molecular subclasses of high-grade glioma predict prognosis, delineate a pattern of disease progression, and resemble stages in neurogenesis. Cancer Cell (2006) 9(3):157–73. 10.1016/j.ccr.2006.02.019 16530701

[9] Qazi MA Vora P Venugopal C Sidhu SS Moffat J Swanton C Intratumoral heterogeneity: pathways to treatment resistance and relapse in human glioblastoma. Ann Oncol (2017) 28(7):1448–56. 10.1093/annonc/mdx169 28407030

[10] Parsons DW Jones S Zhang X Lin JC Leary RJ Angenendt P An integrated genomic analysis of human glioblastoma multiforme. Science (2008) 321(5897):1807–12. 10.1126/science.1164382 PMC2820389 18772396

[11] Cancer Genome Atlas Research N Comprehensive genomic characterization defines human glioblastoma genes and core pathways. Nature (2008) 455(7216):1061–8. 10.1038/nature07385 PMC2671642 18772890

[12] Bunn RC Fowlkes JL Insulin-like growth factor binding protein proteolysis. Trends Endocrinol Metab (2003) 14(4):176–81. 10.1016/s1043-2760(03)00049-3 12714278

[13] Jin L Shen F Weinfeld M Sergi C Insulin Growth Factor Binding Protein 7 (IGFBP7)-Related Cancer and IGFBP3 and IGFBP7 Crosstalk. Front Oncol (2020) 10:727:727. 10.3389/fonc.2020.00727 32500027 PMC7242731

[14] Allard JB Duan C IGF-Binding Proteins: Why Do They Exist and Why Are There So Many? Front Endocrinol (Lausanne) (2018) 9:117:117. 10.3389/fendo.2018.00117 29686648 PMC5900387

[15] Oliva CR Halloran B Hjelmeland AB Vazquez A Bailey SM Sarkaria JN IGFBP6 controls the expansion of chemoresistant glioblastoma through paracrine IGF2/IGF-1R signaling. Cell Commun Signal (2018) 16(1):61. 10.1186/s12964-018-0273-7 30231881 PMC6148802

[16] Hakuno F Takahashi SI IGF1 receptor signaling pathways. J Mol Endocrinol (2018) 61(1):T69–86. 10.1530/JME-17-0311 29535161

[17] Bowers LW Rossi EL O’Flanagan CH deGraffenried LA Hursting SD The Role of the Insulin/IGF System in Cancer: Lessons Learned from Clinical Trials and the Energy Balance-Cancer Link. Front Endocrinol (Lausanne) (2015) 6:77:77. 10.3389/fendo.2015.00077 26029167 PMC4432799

[18] Sachdev D Yee D The IGF system and breast cancer. Endocr Relat Cancer (2001) 8(3):197–209. 10.1677/erc.0.0080197 11566611

[19] Vigneri PG Tirrò E Pennisi MS Massimino M Stella S Romano C The Insulin/IGF System in Colorectal Cancer Development and Resistance to Therapy. Front Oncol (2015) 5:230:230. 10.3389/fonc.2015.00230 26528439 PMC4606066

[20] Yu H Rohan T Role of the insulin-like growth factor family in cancer development and progression. J Natl Cancer Inst (2000) 92(18):1472–89. 10.1093/jnci/92.18.1472 10995803

[21] Pearson JRD Regad T Targeting cellular pathways in glioblastoma multiforme. Signal Transd Targ Ther (2017) 2:17040. 10.1038/sigtrans.2017.40 PMC5661637 29263927

[22] Hirano H Lopes MB Laws ER Jr. Asakura T Goto M Carpenter JE Insulin-like growth factor-1 content and pattern of expression correlates with histopathologic grade in diffusely infiltrating astrocytomas. Neuro Oncol (1999) 1(2):109–19. 10.1093/neuonc/1.2.109 PMC1920755 11550306

[23] Maris C D’Haene N Trepant AL Le Mercier M Sauvage S Allard J IGF-IR: a new prognostic biomarker for human glioblastoma. Br J Cancer (2015) 113(5):729–37. 10.1038/bjc.2015.242 PMC4559821 26291053

[24] Soroceanu L Kharbanda S Chen R Soriano RH Aldape K Misra A Identification of IGF2 signaling through phosphoinositide-3-kinase regulatory subunit 3 as a growth-promoting axis in glioblastoma. Proc Natl Acad Sci U.S.A. (2007) 104(9):3466–71. 10.1073/pnas.0611271104 PMC1802005 17360667

[25] Suvasini R Shruti B Thota B Shinde SV Friedmann-Morvinski D Nawaz Z Insulin growth factor-2 binding protein 3 (IGF2BP3) is a glioblastoma-specific marker that activates phosphatidylinositol 3-kinase/mitogen-activated protein kinase (PI3K/MAPK) pathways by modulating IGF-2. J Biol Chem (2011) 286(29):25882–90. 10.1074/jbc.M110.178012 PMC3138258 21613208

[26] Yin S Girnita A Stromberg T Khan Z Andersson S Zheng H Targeting the insulin-like growth factor-1 receptor by picropodophyllin as a treatment option for glioblastoma. Neuro Oncol (2010) 12(1):19–27. 10.1093/neuonc/nop008 20150364 PMC2940558

[27] Holmes KM Annala M Chua CY Dunlap SM Liu Y Hugen N Insulin-like growth factor-binding protein 2-driven glioma progression is prevented by blocking a clinically significant integrin, integrin-linked kinase, and NF-kappaB network. Proc Natl Acad Sci U.S.A. (2012) 109(9):3475–80. 10.1073/pnas.1120375109 PMC3295320 22345562

[28] Sallinen SL Sallinen PK Haapasalo HK Helin HJ Helen PT Schraml P Identification of differentially expressed genes in human gliomas by DNA microarray and tissue chip techniques. Cancer Res (2000) 60(23):6617–22. 11118044

[29] Santosh V Arivazhagan A Sreekanthreddy P Srinivasan H Thota B Srividya MR Grade-specific expression of insulin-like growth factor-binding proteins-2, -3, and -5 in astrocytomas: IGFBP-3 emerges as a strong predictor of survival in patients with newly diagnosed glioblastoma. Cancer Epidemiol Biomarkers Prev (2010) 19(6):1399–408. 10.1158/1055-9965.EPI-09-1213 20501753

[30] Lin Y Jiang T Zhou K Xu L Chen B Li G Plasma IGFBP-2 levels predict clinical outcomes of patients with high-grade gliomas. Neuro Oncol (2009) 11(5):468–76. 10.1215/15228517-2008-114 PMC2765337 19164435

[31] McDonald KL O’Sullivan MG Parkinson JF Shaw JM Payne CA Brewer JM IQGAP1 and IGFBP2: valuable biomarkers for determining prognosis in glioma patients. J Neuropathol Exp Neurol (2007) 66(5):405–17. 10.1097/nen.0b013e31804567d7 17483698

[32] Wang H Wang H Shen W Huang H Hu L Ramdas L Insulin-like growth factor binding protein 2 enhances glioblastoma invasion by activating invasion-enhancing genes. Cancer Res (2003) 63(15):4315–21. 12907597

[33] Jiang R Mircean C Shmulevich I Cogdell D Jia Y Tabus I Pathway alterations during glioma progression revealed by reverse phase protein lysate arrays. Proteomics (2006) 6(10):2964–71. 10.1002/pmic.200500555 16619307

[34] Praveen Kumar VR Sehgal P Thota B Patil S Santosh V Kondaiah P Insulin like growth factor binding protein 4 promotes GBM progression and regulates key factors involved in EMT and invasion. J Neurooncol (2014) 116(3):455–64. 10.1007/s11060-013-1324-y 24395346

[35] Bei Y Huang Q Shen J Shi J Shen C Xu P IGFBP6 Regulates Cell Apoptosis and Migration in Glioma. Cell Mol Neurobiol (2017) 37(5):889–98. 10.1007/s10571-016-0426-4 PMC11482070 27650075

[36] Schlenska-Lange A Knupfer H Lange TJ Kiess W Knupfer M Cell proliferation and migration in glioblastoma multiforme cell lines are influenced by insulin-like growth factor I in vitro. Anticancer Res (2008) 28(2A):1055–60. 18507054

[37] Hagerstrand D Lindh MB Pena C Garcia-Echeverria C Nister M Hofmann F PI3K/PTEN/Akt pathway status affects the sensitivity of high-grade glioma cell cultures to the insulin-like growth factor-1 receptor inhibitor NVP-AEW541. Neuro Oncol (2010) 12(9):967–75. 10.1093/neuonc/noq029 PMC2940698 20378689

[38] Osuka S Sampetrean O Shimizu T Saga I Onishi N Sugihara E IGF1 receptor signaling regulates adaptive radioprotection in glioma stem cells. Stem Cells (2013) 31(4):627–40. 10.1002/stem.1328 23335250

[39] Osuka S Van Meir EG Overcoming therapeutic resistance in glioblastoma: the way forward. J Clin Invest (2017) 127(2):415–26. 10.1172/JCI89587 PMC5272196 28145904

[40] Zhang M Liu J Li M Zhang S Lu Y Liang Y Insulin-like growth factor 1/insulin-like growth factor 1 receptor signaling protects against cell apoptosis through the PI3K/AKT pathway in glioblastoma cells. Exp Ther Med (2018) 16(2):1477–82. 10.3892/etm.2018.6336 PMC6090237 30116397

[41] Pei Z Lee KC Khan A Wang HY Hyperactivated Insulin Signaling Cascade in Human Glioblastoma Cells. Crit Rev Oncog (2019) 24(3):243–50. 10.1615/CritRevOncog.2019031365 32422022

[42] Gorgisen G Yaren Z Insulin receptor substrate 1 overexpression promotes survival of glioblastoma cells through AKT1 activation. Folia Neuropathol (2020) 58(1):38–44. 10.5114/fn.2020.94005 32337956

[43] Buck E Gokhale PC Koujak S Brown E Eyzaguirre A Tao N Compensatory insulin receptor (IR) activation on inhibition of insulin-like growth factor-1 receptor (IGF-1R): rationale for cotargeting IGF-1R and IR in cancer. Mol Cancer Ther (2010) 9(10):2652–64. 10.1158/1535-7163.MCT-10-0318 20924128

[44] Zhang H Pelzer AM Kiang DT Yee D Down-regulation of type I insulin-like growth factor receptor increases sensitivity of breast cancer cells to insulin. Cancer Res (2007) 67(1):391–7. 10.1158/0008-5472.CAN-06-1712 17210722

[45] Gong Y Ma Y Sinyuk M Loganathan S Thompson RC Sarkaria JN Insulin-mediated signaling promotes proliferation and survival of glioblastoma through Akt activation. Neuro Oncol (2016) 18(1):48–57. 10.1093/neuonc/nov096 26136493 PMC4677408

[46] Liu C Zhang Z Tang H Jiang Z You L Liao Y Crosstalk between IGF-1R and other tumor promoting pathways. Curr Pharm Des (2014) 20(17):2912–21. 10.2174/13816128113199990596 23944361

[47] Manzella L Massimino M Stella S Tirrò E Pennisi MS Martorana F Activation of the IGF Axis in Thyroid Cancer: Implications for Tumorigenesis and Treatment. Int J Mol Sci (2019) 20(13):3258–76. 10.3390/ijms20133258 PMC6651760 31269742

[48] Mauro L Naimo GD Ricchio E Panno ML Ando S Cross-Talk between Adiponectin and IGF-IR in Breast Cancer. Front Oncol (2015) 5:157:157. 10.3389/fonc.2015.00157 26236690 PMC4502352

[49] Vella V Malaguarnera R Nicolosi ML Palladino C Spoleti C Massimino M Discoidin domain receptor 1 modulates insulin receptor signaling and biological responses in breast cancer cells. Oncotarget (2017) 8(26):43248–70. 10.18632/oncotarget.18020 PMC5522143 28591735

[50] Vella V Nicolosi ML Cantafio P Massimino M Lappano R Vigneri P DDR1 regulates thyroid cancer cell differentiation via IGF-2/IR-A autocrine signaling loop. Endocr Relat Cancer (2019) 26(1):197–214. 10.1530/ERC-18-0310 30121624

[51] Hsieh A Ellsworth R Hsieh D Hedgehog/GLI1 regulates IGF dependent malignant behaviors in glioma stem cells. J Cell Physiol (2011) 226(4):1118–27. 10.1002/jcp.22433 20857406

[52] Santoni M Burattini L Nabissi M Morelli MB Berardi R Santoni G Essential role of Gli proteins in glioblastoma multiforme. Curr Protein Pept Sci (2013) 14(2):133–40. 10.2174/1389203711314020005 23544423

[53] Ma Y Tang N Thompson RC Mobley BC Clark SW Sarkaria JN InsR/IGF1R Pathway Mediates Resistance to EGFR Inhibitors in Glioblastoma. Clin Cancer Res (2016) 22(7):1767–76. 10.1158/1078-0432.CCR-15-1677 PMC4818693 26561558

[54] Chua CY Liu Y Granberg KJ Hu L Haapasalo H Annala MJ IGFBP2 potentiates nuclear EGFR-STAT3 signaling. Oncogene (2016) 35(6):738–47. 10.1038/onc.2015.131 PMC4615268 25893308

[55] Fukushima T Kataoka H Roles of insulin-like growth factor binding protein-2 (IGFBP-2) in glioblastoma. Anticancer Res (2007) 27(6A):3685–92. 17970029

[56] Mendes KN Wang GK Fuller GN Zhang W JNK mediates insulin-like growth factor binding protein 2/integrin alpha5-dependent glioma cell migration. Int J Oncol (2010) 37(1):143–53. 10.3892/ijo_00000662 20514406

[57] Thota B Arimappamagan A Kandavel T Shastry AH Pandey P Chandramouli BA STAT-1 expression is regulated by IGFBP-3 in malignant glioma cells and is a strong predictor of poor survival in patients with glioblastoma. J Neurosurg (2014) 121(2):374–83. 10.3171/2014.4.JNS131198 24878287

[58] Chen CH Chen PY Lin YY Feng LY Chen SH Chen CY Suppression of tumor growth via IGFBP3 depletion as a potential treatment in glioma. J Neurosurg (2019) 132(1):168–79. 10.3171/2018.8.JNS181217 30641835

[59] Moreno MJ Ball M Andrade MF McDermid A Stanimirovic DB Insulin-like growth factor binding protein-4 (IGFBP-4) is a novel anti-angiogenic and anti-tumorigenic mediator secreted by dibutyryl cyclic AMP (dB-cAMP)-differentiated glioblastoma cells. Glia (2006) 53(8):845–57. 10.1002/glia.20345 16586492

[60] Dong C Zhang J Fang S Liu F IGFBP5 increases cell invasion and inhibits cell proliferation by EMT and Akt signaling pathway in Glioblastoma multiforme cells. Cell Div (2020) 15:4. 10.1186/s13008-020-00061-6 32127912 PMC7047354

[61] Banelli B Forlani A Allemanni G Morabito A Pistillo MP Romani M MicroRNA in Glioblastoma: An Overview. Int J Genomics (2017) 2017:7639084. 10.1155/2017/7639084 29234674 PMC5695025

[62] Luo JW Wang X Yang Y Mao Q Role of micro-RNA (miRNA) in pathogenesis of glioblastoma. Eur Rev Med Pharmacol Sci (2015) 19(9):1630–9. 26004603

[63] Wang H Tang C Na M Ma W Jiang Z Gu Y miR-422a Inhibits Glioma Proliferation and Invasion by Targeting IGF1 and IGF1R. Oncol Res (2017) 25(2):187–94. 10.3727/096504016X14732772150389 PMC7840806 28277190

[64] Chen PH Cheng CH Shih CM Ho KH Lin CW Lee CC The Inhibition of microRNA-128 on IGF-1-Activating mTOR Signaling Involves in Temozolomide-Induced Glioma Cell Apoptotic Death. PloS One (2016) 11(11):e0167096. 10.1371/journal.pone.0167096 27893811 PMC5125683

[65] Guo T Feng Y Liu Q Yang X Jiang T Chen Y MicroRNA-320a suppresses in GBM patients and modulates glioma cell functions by targeting IGF-1R. Tumour Biol (2014) 35(11):11269–75. 10.1007/s13277-014-2283-4 25117070

[66] He Z Cen D Luo X Li D Li P Liang L Downregulation of miR-383 promotes glioma cell invasion by targeting insulin-like growth factor 1 receptor. Med Oncol (2013) 30(2):557. 10.1007/s12032-013-0557-0 23564324

[67] Jiang J Wang W Fang D Jin X Ding L Sun X MicroRNA186 targets IGF1R and exerts tumorsuppressing functions in glioma. Mol Med Rep (2017) 16(5):7821–8. 10.3892/mmr.2017.7586 28944896

[68] Shi H Yang H Xu S Zhao Y Liu J miR-505 functions as a tumor suppressor in glioma by targeting insulin like growth factor 1 receptor expression. Int J Clin Exp Pathol (2018) 11(9):4405–13. PMC6962946 31949837

[69] Shi ZM Wang XF Qian X Tao T Wang L Chen QD MiRNA-181b suppresses IGF-1R and functions as a tumor suppressor gene in gliomas. RNA (2013) 19(4):552–60. 10.1261/rna.035972.112 PMC3677265 23431408

[70] Wang B Sun F Dong N Sun Z Diao Y Zheng C MicroRNA-7 directly targets insulin-like growth factor 1 receptor to inhibit cellular growth and glucose metabolism in gliomas. Diagn Pathol (2014) 9:211. 10.1186/s13000-014-0211-y 25394492 PMC4236426

[71] Wang J Liu H Tian L Wang F Han L Zhang W miR-15b Inhibits the Progression of Glioblastoma Cells Through Targeting Insulin-like Growth Factor Receptor 1. Horm Cancer (2017) 8(1):49–57. 10.1007/s12672-016-0276-z 27896672 PMC10355966

[72] Yang HA Wang X Ding F Pang Q MiRNA-323-5p Promotes U373 Cell Apoptosis by Reducing IGF-1R. Med Sci Monit (2015) 21:3880–6. 10.12659/msm.895037 PMC4681375 26656446

[73] Zhang Y Chen X Lian H Liu J Zhou B Han S MicroRNA-503 acts as a tumor suppressor in glioblastoma for multiple antitumor effects by targeting IGF-1R. Oncol Rep (2014) 31(3):1445–52. 10.3892/or.2013.2951 PMC4440219 24378652

[74] Ramakrishnan V Xu B Akers J Nguyen T Ma J Dhawan S Radiation-induced extracellular vesicle (EV) release of miR-603 promotes IGF1-mediated stem cell state in glioblastomas. EBioMedicine (2020) 55:102736. 10.1016/j.ebiom.2020.102736 32361246 PMC7195524

[75] Chen PH Chang CK Shih CM Cheng CH Lin CW Lee CC The miR-204-3p-targeted IGFBP2 pathway is involved in xanthohumol-induced glioma cell apoptotic death. Neuropharmacology (2016) 110(Pt A):362–75. 10.1016/j.neuropharm.2016.07.038 27487563

[76] Li X Liu Y Granberg KJ Wang Q Moore LM Ji P Two mature products of MIR-491 coordinate to suppress key cancer hallmarks in glioblastoma. Oncogene (2015) 34(13):1619–28. 10.1038/onc.2014.98 PMC4205227 24747968

[77] Lee CC Chen PH Ho KH Shih CM Cheng CH Lin CW The microRNA-302b-inhibited insulin-like growth factor-binding protein 2 signaling pathway induces glioma cell apoptosis by targeting nuclear factor IA. PloS One (2017) 12(3):e0173890. 10.1371/journal.pone.0173890 28323865 PMC5360322

[78] Xu J Liao X Lu N Liu W Wong CW Chromatin-modifying drugs induce miRNA-153 expression to suppress Irs-2 in glioblastoma cell lines. Int J Cancer (2011) 129(10):2527–31. 10.1002/ijc.25917 21213215

[79] Kefas B Godlewski J Comeau L Li Y Abounader R Hawkinson M microRNA-7 inhibits the epidermal growth factor receptor and the Akt pathway and is down-regulated in glioblastoma. Cancer Res (2008) 68(10):3566–72. 10.1158/0008-5472.CAN-07-6639 18483236

[80] Huo L Wang B Zheng M Zhang Y Xu J Yang G miR-128-3p inhibits glioma cell proliferation and differentiation by targeting NPTX1 through IRS-1/PI3K/AKT signaling pathway. Exp Ther Med (2019) 17(4):2921–30. 10.3892/etm.2019.7284 PMC6425241 30906475

[81] Chen KC Chen PH Ho KH Shih CM Chou CM Cheng CH IGF-1-enhanced miR-513a-5p signaling desensitizes glioma cells to temozolomide by targeting the NEDD4L-inhibited Wnt/beta-catenin pathway. PloS One (2019) 14(12):e0225913. 10.1371/journal.pone.0225913 31805126 PMC6894868

[82] Yang CH Yue J Pfeffer SR Fan M Paulus E Hosni-Ahmed A MicroRNA-21 promotes glioblastoma tumorigenesis by down-regulating insulin-like growth factor-binding protein-3 (IGFBP3). J Biol Chem (2014) 289(36):25079–87. 10.1074/jbc.M114.593863 PMC4155674 25059666

[83] Denduluri SK Idowu O Wang Z Liao Z Yan Z Mohammed MK Insulin-like growth factor (IGF) signaling in tumorigenesis and the development of cancer drug resistance. Genes Dis (2015) 2(1):13–25. 10.1016/j.gendis.2014.10.004 25984556 PMC4431759

[84] Zhu D Tu M Zeng B Cai L Zheng W Su Z Up-regulation of miR-497 confers resistance to temozolomide in human glioma cells by targeting mTOR/Bcl-2. Cancer Med (2017) 6(2):452–62. 10.1002/cam4.987 PMC5313645 28064447

[85] Tanksley JP Chen X Coffey RJ NEDD4L is downregulated in colorectal cancer and inhibits canonical WNT signaling. PloS One (2013) 8(11):e81514. 10.1371/journal.pone.0081514 24312311 PMC3842946

[86] He S Deng J Li G Wang B Cao Y Tu Y Down-regulation of Nedd4L is associated with the aggressive progression and worse prognosis of malignant glioma. Jpn J Clin Oncol (2012) 42(3):196–201. 10.1093/jjco/hyr195 22217575

[87] Bao S Wu Q McLendon RE Hao Y Shi Q Hjelmeland AB Glioma stem cells promote radioresistance by preferential activation of the DNA damage response. Nature (2006) 444(7120):756–60. 10.1038/nature05236 17051156

[88] Simpson AD Soo YWJ Rieunier G Aleksic T Ansorge O Jones C Type 1 IGF receptor associates with adverse outcome and cellular radioresistance in paediatric high-grade glioma. Br J Cancer (2020) 122(5):624–9. 10.1038/s41416-019-0677-1 PMC7054265 31857716

[89] Brennan CW Verhaak RG McKenna A Campos B Noushmehr H Salama SR The somatic genomic landscape of glioblastoma. Cell (2013) 155(2):462–77. 10.1016/j.cell.2013.09.034 PMC3910500 24120142

[90] Saleem H Kulsoom Abdul U Kucukosmanoglu A Houweling M Cornelissen FMG Heiland DH The TICking clock of EGFR therapy resistance in glioblastoma: Target Independence or target Compensation. Drug Resist Update (2019) 43:29–37. 10.1016/j.drup.2019.04.002 31054489

[91] Chakravarti A Loeffler JS Dyson NJ Insulin-like growth factor receptor I mediates resistance to anti-epidermal growth factor receptor therapy in primary human glioblastoma cells through continued activation of phosphoinositide 3-kinase signaling. Cancer Res (2002) 62(1):200–7. 11782378

[92] Alamdari-Palangi V Amini R Karami H MiRNA-7 enhances erlotinib sensitivity of glioblastoma cells by blocking the IRS-1 and IRS-2 expression. J Pharm Pharmacol (2020) 72(4):531–8. 10.1111/jphp.13226 32026479

[93] Drappatz J Norden AD Wen PY Therapeutic strategies for inhibiting invasion in glioblastoma. Expert Rev Neurother (2009) 9(4):519–34. 10.1586/ern.09.10 19344303

[94] Verhaak RG Hoadley KA Purdom E Wang V Qi Y Wilkerson MD Integrated genomic analysis identifies clinically relevant subtypes of glioblastoma characterized by abnormalities in PDGFRA, IDH1, EGFR, and NF1. Cancer Cell (2010) 17(1):98–110. 10.1016/j.ccr.2009.12.020 20129251 PMC2818769

[95] Almiron Bonnin DA Ran C Havrda MC Liu H Hitoshi Y Zhang Z Insulin-Mediated Signaling Facilitates Resistance to PDGFR Inhibition in Proneural hPDGFB-Driven Gliomas. Mol Cancer Ther (2017) 16(4):705–16. 10.1158/1535-7163.MCT-16-0616 28138037

[96] Pyonteck SM Akkari L Schuhmacher AJ Bowman RL Sevenich L Quail DF CSF-1R inhibition alters macrophage polarization and blocks glioma progression. Nat Med (2013) 19(10):1264–72. 10.1038/nm.3337 PMC3840724 24056773

[97] Ries CH Cannarile MA Hoves S Benz J Wartha K Runza V Targeting tumor-associated macrophages with anti-CSF-1R antibody reveals a strategy for cancer therapy. Cancer Cell (2014) 25(6):846–59. 10.1016/j.ccr.2014.05.016 24898549

[98] Quail DF Bowman RL Akkari L Quick ML Schuhmacher AJ Huse JT The tumor microenvironment underlies acquired resistance to CSF-1R inhibition in gliomas. Science (2016) 352(6288):aad3018. 10.1126/science.aad3018 27199435 PMC5450629

[99] Rodvold JJ Xian S Nussbacher J Tsui B Cameron Waller T Searles SC IRE1alpha and IGF signaling predict resistance to an endoplasmic reticulum stress-inducing drug in glioblastoma cells. Sci Rep (2020) 10(1):8348. 10.1038/s41598-020-65320-6 32433555 PMC7239929

[100] Tirrò E Martorana F Romano C Vitale SR Motta G Di Gregorio S Molecular Alterations in Thyroid Cancer: From Bench to Clinical Practice. Genes (Basel) (2019) 10(9):709–41. 10.3390/genes10090709 PMC6771012 31540307

[101] Massimino M Tirro E Stella S Frasca F Vella V Sciacca L Effect of Combined Epigenetic Treatments and Ectopic NIS Expression on Undifferentiated Thyroid Cancer Cells. Anticancer Res (2018) 38(12):6653–62. 10.21873/anticanres.13032 30504373

[102] Tirrò E Massimino M Romano C Pennisi MS Stella S Vitale SR Chk1 Inhibition Restores Inotuzumab Ozogamicin Citotoxicity in CD22-Positive Cells Expressing Mutant p53. Front Oncol (2019) 9:57:57. 10.3389/fonc.2019.00057 30834235 PMC6387953

[103] Tirrò E Massimino M Stella S Zammit V Consoli ML Pennisi MS Efficacy of Nilotinib in a CML Patient Expressing the Three-way Complex Variant Translocation t ( ). Anticancer Res (2019) 39(7):3893–9. 10.21873/anticanres.13540 31262918

[104] Andrews DW Resnicoff M Flanders AE Kenyon L Curtis M Merli G Results of a pilot study involving the use of an antisense oligodeoxynucleotide directed against the insulin-like growth factor type I receptor in malignant astrocytomas. J Clin Oncol (2001) 19(8):2189–200. 10.1200/JCO.2001.19.8.2189 11304771

[105] Trojan J Cloix JF Ardourel MY Chatel M Anthony DD Insulin-like growth factor type I biology and targeting in malignant gliomas. Neuroscience (2007) 145(3):795–811. 10.1016/j.neuroscience.2007.01.021 17320297

[106] Ly A Duc HT Kalamarides M Trojan LA Pan Y Shevelev A Human glioma cells transformed by IGF-I triple helix technology show immune and apoptotic characteristics determining cell selection for gene therapy of glioblastoma. Mol Pathol (2001) 54(4):230–9. 10.1136/mp.54.4.230 PMC1187073 11477137

[107] Andrews DW Garcia S Judy KD Harshyne LA Govindarajan S Kenyon L Abstract CT038: Results of a Phase Ib trial of an autologous cell vaccine for newly diagnosed glioblastoma. Cancer Res (2019) 79:CT038–8. 10.1158/1538-7445.SABCS18-CT038

[108] Zhou X Shen F Ma P Hui H Pei S Chen M GSK1838705A, an IGF-1R inhibitor, inhibits glioma cell proliferation and suppresses tumor growth in vivo. Mol Med Rep (2015) 12(4):5641–6. 10.3892/mmr.2015.4129 PMC4581800 26238593

[109] Zhou X Zhao X Li X Ping G Pei S Chen M PQ401, an IGF-1R inhibitor, induces apoptosis and inhibits growth, proliferation and migration of glioma cells. J Chemother (2016) 28(1):44–9. 10.1179/1973947815Y.0000000026 25971682

[110] Premkumar DR Jane EP Pollack IF Co-administration of NVP-AEW541 and dasatinib induces mitochondrial-mediated apoptosis through Bax activation in malignant human glioma cell lines. Int J Oncol (2010) 37(3):633–43. 10.3892/ijo_00000712 20664932

[111] Simpson A Petnga W Macaulay VM Weyer-Czernilofsky U Bogenrieder T Insulin-Like Growth Factor (IGF) Pathway Targeting in Cancer: Role of the IGF Axis and Opportunities for Future Combination Studies. Targ Oncol (2017) 12(5):571–97. 10.1007/s11523-017-0514-5 PMC5610669 28815409

[112] Zamykal M Martens T Matschke J Gunther HS Kathagen A Schulte A Inhibition of intracerebral glioblastoma growth by targeting the insulin-like growth factor 1 receptor involves different context-dependent mechanisms. Neuro Oncol (2015) 17(8):1076–85. 10.1093/neuonc/nou344 PMC4490867 25543125

[113] Fouladi M Perentesis JP Wagner LM Vinks AA Reid JM Ahern C A Phase I Study of Cixutumumab (IMC-A12) in Combination with Temsirolimus (CCI-779) in Children with Recurrent Solid Tumors: A Children’s Oncology Group Phase I Consortium Report. Clin Cancer Res (2015) 21(7):1558–65. 10.1158/1078-0432.CCR-14-0595 PMC4454739 25467181

